# Stressful Life Events, Cognitive Biases, and Symptoms of Depression in Young Adults

**DOI:** 10.3389/fpsyg.2019.02165

**Published:** 2019-09-20

**Authors:** Władysław Łosiak, Agata Blaut, Joanna Kłosowska, Julia Łosiak-Pilch

**Affiliations:** ^1^Department of Philosophy, Institute of Psychology, Jagiellonian University, Kraków, Poland; ^2^Department of Philosophy, Institute of Pedagogy, Jagiellonian University, Kraków, Poland

**Keywords:** symptoms of depression, rumination, memory bias, emotional attentional blink, stressful life events

## Abstract

Although the link between stressful experiences and depression has been supported in numerous studies, the specific mechanisms of this relationship are still unclear. Cognitive theories of depression postulate that the influence of stress on depression may be modified by cognitive factors. The aim of the present study was to examine the interplay between negative life events, cognitive vulnerability factors, and depressive symptoms. It was hypothesized that the relationship between negative life events and symptoms of depression is shaped by rumination and cognitive biases. The study sample consisted of 108 young adults (19 men and 89 women; *M* = 20.31; *SD* = 1.84). Memory bias and attentional bias were assessed using the Attentional Blink Task and the Memory Task, respectively. Rumination and depressive symptoms were assessed via self-report questionnaires. Logistic regression and moderation analyses were conducted to examine the relationship between the study variables. Stressful life events, rumination and memory bias were found to be significantly related to depressive symptoms. Moderation analyses revealed that there is a positive relationship between negative life events and depressive symptoms but only among individuals characterized by an elevated level of rumination and among participants exhibiting negative attentional bias. The results provide further evidence for cognitive models of depression.

## Introduction

Psychological factors that are considered to be risk factors for depression are negative experiences associated with stressful life events, the individual tendency to ruminate, and other cognitive vulnerability factors such as irrational beliefs, maladaptive schemas, or cognitive biases. Numerous studies (e.g., Brown and Harris, 1986; Post, 1992; Williams et al., 1997; Thomsen, 2006; Nolen-Hoeksema et al., 2007; Riggs and Han, 2009; Marks et al., 2010; Eberhart et al., 2011; Kircanski et al., 2012) have shown that these factors play an important role in the development of depression symptoms.

In many research dating back to the 1980s (Brown and Harris, 1986; Riggs and Han, 2009; Marks et al., 2010; Eberhart et al., 2011), the occurrence of depressive symptoms in the non-clinical population was found to be related to stressful experiences. Similar results were obtained in studies focused on adolescents and young adults (Johnson et al., 2012; Maughan et al., 2013; Lester, 2014; Herbison et al., 2017). According to the “diathesis–stress” model (e.g., Ingram and Luxton, 2005), symptoms of depression develop as a result of life stress only in individuals who have pre-existing vulnerabilities. When the concept of rumination – defined as a specific response style that increases the incidence, severity and duration of depressive states – was formulated (Nolen-Hoeksema, 1991), ruminative tendency began to be considered as an important factor that increases the risk of depression (see Thomsen, 2006, for a review) and a predictor of depressive symptoms in non-clinical populations (e.g., Nolen-Hoeksema et al., 2007). The results of recent studies have suggested that rumination may interact with stress and thus increase depression symptoms (Skitch and Abela, 2008; Abela and Hankin, 2011; Bastin et al., 2015; Hamlat et al., 2015; Padilla Paredes and Calvete Zumalde, 2015; Connolly and Alloy, 2017; Shapero et al., 2017).

On the other hand, there are numerous opinions that depression may be related to the co-occurrence of stress and attentional, memory-related and interpretational cognitive biases, among others. Cognitive theories of depression postulate that processing biases are vulnerability and maintenance factors for this disorder (Beck, 1976; Williams et al., 1997; Beevers, 2005; Mathews and MacLeod, 2005; Kircanski et al., 2012). According to Beck’s model, these negative views of self and the world (schemas) are formed in childhood and adolescence and remain latent until activated by stressful life events (Beck, 1976). Beevers (2005), in his dual process model, distinguishes two modes of information that process underlying cognitive vulnerability to depression: the associative mode, which is automatic, quick and effortless, and the reflective mode, which is controlled, conscious and rational. The model stresses the role of the interplay between these two processing modes: vulnerability to depression may occur when negative associative processing is not corrected by reflective processing.

Many studies have shown that depression is associated with biased memory processing, which is also a hallmark feature in cognitive models of depression (Williams et al., 1997; Kircanski et al., 2012). In particular, according to a meta-analysis of mood-congruent memory in depression, sub-clinical depression is associated with a lack of positive recall asymmetry that is often found in the non-depressed (Matt et al., 1992).

Other studies have shown that depression is characterized by an attentional bias for negative and self-referential information (for review, see De Raedt and Koster, 2010; Gotlib and Joormann, 2010; Peckham et al., 2010; Disner et al., 2011). According to some authors, attentional bias in depression reflects sustained attention, difficulty in disengagement, or other forms of interference occurring at later more elaborative stages of processing (Gotlib et al., 2004b; Koster et al., 2005). Recent eye-tracking studies on attentional disengagement have also shown that both clinically and subclinically depressed individuals are characterized by difficulties with inhibiting the processing of negative material when prompted to move their gaze away from it (Sanchez et al., 2013; Sanchez et al., 2017). In contrast to the spatial visual attention studies described above, research on temporal characteristics of attentional bias in depression have not been extensive (Koster et al., 2009; Romens et al., 2011; Milders et al., 2016). Attentional blink (AB) is a widely studied phenomenon that reflects the temporal costs of allocating selective attention. It is typically studied using rapid serial visual presentation (RSVP) tasks in which participants often fail to detect a second salient target occurring in succession if it is presented between 180 and 450 ms after the first one. There is growing evidence (see McHugo et al., 2013 for a review) that the RSVP task and the EAB (emotional attentional blink) are useful as measures of attentional biases to relevant stimuli in psychopathology studies. For example, in one such study Koster et al. (2009) found that accuracy of target identification was significantly impaired when it was preceded by a negative word in a depressive group. This effect was only found when T2 was presented in close temporal proximity to T1 (Lag 2, 200 ms), which may indicate that the attentional blink effect is stronger when depressive participants report negative stimuli.

There have been many attempts to establish the nature of the relationship between cognitive factors, negative events and symptoms of depression. However, it is worth noting that almost all such studies used self-report measures of cognitive processes (e.g., Stange et al., 2013, 2014; Bastin et al., 2015; Shapero et al., 2017). Implementing such measures in depression research may increase the possibility of negative response bias. Also, according to Beevers (2005) self-report questionnaires can only measure cognitive products that result from reflective processing, but cognitive vulnerability depends largely on automatic processes that are typically not available for consciousness. Therefore, it seems important that various cognitive processes are examined in studies on the relationship between stress and depression. This requires using both questionnaires and behavioral methods, including measures based on correctness and response time in computerized tasks.

The results of some investigations suggest that stress, rumination and cognitive bias may all interact with each other and thus increase depression. In their experimental study, Morrison and O’Connor (2008) obtained results suggesting that negative attentional bias shows a trend toward interacting with rumination and stress in predicting dysphoria 3 weeks later. Very similar results were obtained in the longitudinal study by Sanchez-Lopez et al. (2019), who examined how baseline negative attention bias and high habitual ruminative responses cooperate with the subsequent occurrence of perceived stress to predict depression. They found that depression increases across time, especially in the case of individuals who reported a higher degree of adverse events and who were characterized simultaneously by both longer times when disengaging attention from negative information and higher ruminative brooding levels at the baseline assessment. Although the results of both of these studies did not reach the level of statistical significance, they describe similar effects which seem to be worth further exploration.

The purpose of the study was to examine the interplay between stressful life events, cognitive biases, habitual rumination and symptoms of depression in a non-clinical sample of young adults. It was focused on the evaluation of cognitive mechanisms that explain the relationship between stressful events and depression. Cognitive biases that were taken into consideration concerned attention and memory (indicated by attentional blink and memory selectiveness), while a tendency to ruminate was an indicator of reflective processing strategy relevant for depression. The following hypotheses were formulated:

(1) Depressive symptoms are associated with a higher level of stress related to negative life events, as well as higher levels of rumination, negative memory bias, and attentional bias.
(2) The relationship between stress and depressiveness is modified by individual cognitive vulnerability factors: tendency to ruminate, attention and memory biases.

In particular:

(a) Stressful life events are positively related to depressive symptoms only in individuals characterized by an elevated level of rumination.
(b) Stressful life events are positively related to depressive symptoms only in individuals characterized by an elevated level of negative attentional bias.
(c) Stressful life events are positively related to depressive symptoms only in individuals characterized by an elevated level of negative memory bias.

Taking into consideration the initial evidence (Morrison and O’Connor, 2008; Sanchez-Lopez et al., 2019) suggesting that the co-occurrence of negative cognitive biases and high habitual use of rumination in combination with high perceived stress may be a predictor of depression level, the following research question was also formulated:

Do stressful life events, tendency to ruminate and cognitive biases interact in their effect on depression?

## Materials And Methods

### Participants and Procedure

Participants were 108 (19 men and 89 women) university and high-school students aged 18–25 (*M* = 20.31; *SD* = 1.84) who volunteered to participate in the study and gave informed consent. They were recruited from the community using an advertisement among students and snowball sampling. They were informed that the study concerned attention, memory, and mood. They declared that they had not been diagnosed with any disorder and did not use any medications prior to or at the time of the study.

Participants were invited to the sound-attenuated laboratory in groups of six and were placed individually in front of a PC computer with 60 Hz monitor. Items of self-report measures were presented on the screen and they chose the answer by pressing the appropriate key. Tests measuring attention and memory were also presented on the screen and participants had to press the key or write words using the keyboard. The software used in the cognitive tasks was written specifically for this study in C++. All participants were presented materials on the screen in the same order: first, self-report measures of symptoms of depression, followed by rumination tendency and stressful life events, then the procedures testing attention emotional blink and memory biases. The entire test session in the lab took approximately 45 min. No compensation (financial or otherwise) was offered as an incentive to participate. The study protocol, information on the study, informed consent, and related materials were submitted and approved by the ethics committee of the Institute of Psychology, Jagiellonian University in Kraków.

### Measures

#### The Center for Epidemic Depression Scale (CES-D)

The 20-item CES-D (Radloff, 1977) is a self-report scale which assesses depressive symptoms in the general population. Participants report the frequency of symptoms (e.g., I felt gloomy) experienced during the last week on a 4-point rating scale, from 0 – less than one day to 3 – five to seven days. The CES-D provides a total score ranging from 0 to 60. In the current study we utilized the Polish translation of CES-D, which reveals very good internal consistency with Cronbach’s alpha coefficient α = 0.92 that was reported in the validation study (Dojka et al., 2003) as well as in the present study. In the analyses, we used the typically recommended CES-D score of 16 as the cutoff point to indicate cases of depression. Individuals with a score of 16 or more had to have either at least 6 of the 20 symptoms in the CES-D with persistence for most of the previous week, or a majority of the symptoms on the scale for shorter periods of time (Weissman et al., 1977).

#### Rumination Questionnaire

The Rumination Questionnaire (RQ) by Baryła and Wojciszke (2005) measures ruminations about the self and the social world. One of the two subscales of the questionnaire was used which measured individual tendency to ruminate about the self. The subscale consists of 10 items concerning ruminative thoughts (e.g., I cannot stop thinking about my failures) and the participant reported the frequency of a given thought on a five-point rating scale, from 1 – never to 5 – very often. The subscale has good psychometric properties: a Cronbach’s alpha of 0.89 was reported in the validation study (Baryła and Wojciszke, 2005) and α = 0.95 in the current study. The scores of subscales of RQ was found to correlate in the assumed direction with different measures of anxiety, depression, decreased mood, satisfaction of life and self-esteem (Baryła and Wojciszke, 2005).

#### Stressful Life Events Scale

The scale was prepared for the purpose of the study. A list of stressful life events was constructed based on the results of a study in which a group of 65 students (28 men and 37 women) were first asked to name stressful events that had happened to them in the last 6 months, and then to name stressful events that they think happen to young people of their age. As a result the scale contained a list of 45 life events that were grouped according to their content: family (8 events, e.g., divorce of parents), education (5 events, e.g., failing a test), peers (7 events, e.g., losing a friend), health (5 events, e.g., serious illness), close relationships (6 events, e.g., argument with boyfriend/girlfriend), experience of violence (3 events, e.g., being a victim of a crime), legal problems (3 events, e.g., being arrested) and other general problems (8 events, e.g., traffic accident, losing something valuable). Participants of the study were asked to check events that happened to them during the last 6 months and then evaluate them on a five-point scale on which 1 – not very stressful and 5 – very stressful. The 6 months period of assessment was chosen because participants were high-school and university students who had just started their second semester, and we did not want this period to overlap with the holiday break, which is a time that is mostly free of school-related stressors (this is one of the most important sources of stress for young people). Two measures were taken for each participant: the total number of indicated stressful events and a cumulative evaluation of their stressfulness (sum of evaluations of all checked events). Cronbach’s Alpha calculated in the present study was α = 0.88 for the total number of events subscale and α = 0.82 for the impact subscale. Since almost all stressors are dependent on context for their likely severity and people may vary greatly in how they are affected by various negative life events, in further analyses a cumulative index of life events was used that was calculated by multiplying the total number of events by the cumulative evaluation of their stressfulness.

#### Memory Task

The memory task consisted of 30 trials. Each trial of this task began with the presentation of a 500 ms fixation point, after which one of the words from the memory set was shown for 7 s in the middle of the computer screen. Ten words of each valence (negative, positive, neutral) were presented to each participant in a randomized order. To encourage deeper processing of the words, the participants were instructed to try to memorize the words and at the same time rate their valence using the mouse pointer. Encoding was followed by a filler task which lasted about 5 min. Every trial of the filler task started with a 500 ms presentation of a white cross that served as a fixation point. The fixation point was immediately replaced by a randomly chosen word (either “RIGHT” or “LEFT”) presented in the center of the computer screen. The participants were instructed to press the arrow keys in response to the direction dictated by the words. Immediately after the filler task was completed the participants were asked to write down as many memorized words as they could during a 5 min recall period.

A memory bias index was calculated by subtracting the number of correctly recalled positive words from the number of correctly recalled negative words. A positive value of this index indicates that the negative words were recalled better than the positive words.

#### Rapid Serial Visual Presentation (RSVP) Task

The RSVP task was based on the procedure used in Koster et al. (2009). Each of the 135 trials started with a 500 ms fixation point followed by a rapid serial presentation of 13 words, presented for 100 ms each. Every such set consisted of two targets (T1 and T2) printed in green, and 11 neutral white distractors in random order. The participants were instructed to report both targets at the end of each trial by typing them on the computer keyboard. T2 was always neutral and, depending on the condition, T1 was negative, positive, or neutral. T1 was presented in position 3, 4, or 5 and T2 was presented 2 (200 ms), 4 (400 ms), or 6 (600 ms) words after T1. There were 27 different types of trials: T1 valence (negative, positive, and neutral) × T1 position (3, 4, or 5) × T2 lag: (2, 4, or 6). Five trials were administered per trial type resulting in a total number of 135 trials, as in Koster et al.’s (2009) study. However since the data were averaged over the T1 position prior to fitting the models, there were effectively 15 trials per condition.

An attentional bias index was calculated in a way that made it comparable to the memory bias index. It was obtained by subtracting the number of correct identifications in lag 2 condition of T2 when T1 was negative from the number of correct identifications of T2 when T1 was positive. A positive value of this index indicates that the negative material received preferential attentional processing over the positive material.

#### Stimuli

Verbal stimuli used in cognitive tasks consisted of 57 words: 19 neutral (valence: *M* = 0.21, *SD* = 1.02; arousal: *M* = 1.08, *SD* = 0.82), 19 negative (valence: *M* = -1.83, *SD* = 0.92; arousal: *M* = 2.62, *SD* = 1.01) and 19 positive adjectives (valence: *M* = 2.01, *SD* = 0.94; arousal: *M* = 2.73, *SD* = 1.30). The subset of 30 words were used in the memory task and the remaining 27 were used in the RSVP task. Stimuli used in the RSVP task comprised words of three to six characters. The words were selected from previous research (Blaut et al., 2013) and from the Nencki Affective Word List NAWL (Riegel et al., 2015) The words were matched for frequency, length and imageability. The negative words were associated with lowered mood (e.g., sad), self-esteem (e.g., useless) and failure (e.g., hopeless), etc. Another set of 79 neutral words served as distracting words in the RSVP task.

### Data Analysis

Firstly the Shapiro–Wilk’s test was used to assess the distributions of study variables and also to check the skewness and kurtosis for each variable. Secondly the correlational analysis was performed to assess the relationships between variables. Paired-sample *t*-tests were used to assess the attentional blink effect. Next logistic regression analysis was employed to: (1) test which of analyzed variables (rumination/life events/memory bias/attentional bias) are significantly related to depression; (2) check whether rumination and cognitive biases serve (independently and together) as moderators in the relationship between life events and depression. Interaction effects were examined in parallel models. To explore significant interaction effects Johnson-Neyman technique was utilized. All analyses were conducted using SPSS version 22. The Johnson-Neyman procedure was performed using the PROCESS macro for SPSS (Hayes, 2018). Model number 1 (moderation analysis with one moderator) and model number 3 (moderation analysis with two moderators) from PROCESS were utilized. Cases with missing data were excluded from the analyses. Bonferroni correction for multiple comparisons was not used because it could not be assumed that the results of separate analyses were independent.

As our goal was to examine the link between negative life events and cognitive distortions and depressive symptoms that have potential clinical significance, prior to running the analyses the sample was subsequently divided into two subgroups according to the cut-off criteria proposed by Weissman et al. (1977): the “no-depression” subgroup (CESD < 16) and the “depression” subgroup (CESD ≥ 16). “No-depression” group consisted of 52 participants (42 females, 10 males) and “depression” group consisted of 56 participants (47 females, 9 males). These groups did not differ significantly in terms of age [*t*(106) = 1.13, *p* = 0.26, *d* = 0.22] and sex [χ^2^(1) = 0.19, *p* = 0.67, φ = 0.04].

## Results

### Descriptive Statistics and Correlational Analysis

The Shapiro–Wilk test showed that among the study variables, the rumination scale and the indicator variable of attentional bias were normally distributed in the study sample (*p* > 0.05), whereas other measured variables significantly deviated from the norm. Therefore, Spearman’s correlation coefficients were calculated for memory bias and age, and Pearson’s correlation coefficients were calculated for normally distributed variables. Point-biserial correlations were calculated for dichotomized depression variable and sex. The relation between depression and sex was examined using the chi-squared test.

Correlation analysis revealed significant moderate relationships between depression and ruminations as well as between depression and life events. Additionally, ruminations were positively and weakly associated with life events. All correlation coefficients as well as descriptive statistics of study variables are presented in Table 1.

**TABLE 1:** Descriptive statistics and results of correlational analysis.

	**Mean (SD)**	**Skewness (SD)**	**Kurtosis (SD)**	**1**	**2**	**3**	**4**	**5**	**6**
Rumination (1)	22.67 (8.98)	0.16 (0.26)	−0.26(0.51)	1					
Life events (2)	178.53 (214.16)	1.56 (0.26)	1.68 (0.51)	0.24^∗^	1				
Negative attentional bias (3)	−0.00(0.15)	0.07 (0.24)	0.34 (0.47)	0.14	0.09	1			
Negative memory bias (4)	−0.90(1.69)	0.07 (0.26)	−0.35(0.51)	–0.11	–0.14	–0.15	1		
Depression^a^ (5)	–	–	–	0.28^∗∗^	0.40^∗∗∗^	0.16	0.05	1	
Age (6)	20.27 (2.22)	0.85 (0.23)	1.95 (0.46)	–0.07	–0.07	0.10	–0.08	–0.11	1
Sex^b^	–	–	–	–0.04	0.04	–0.12	0.07	0.19^c^	–0.11

*^*a*^Non-depressed (CESD < 16) were coded as 0, depressed (CESD ≥ 16) were coded as 1. ^*b*^Women were coded as 1, men were coded as 2. ^*c*^Results of chi-square test. ^∗^p < 0.05; ^∗∗^p < 0.01; ^∗∗∗^p < 0.001.*

### Attentional Blink Effect

Percentage accuracies were calculated for all conditions (T2 preceded by positive T1 word, negative T1 word, neutral T1 word) and lags (2, 4, or 6 filler words between target words) in the attentional blink task. To verify that an attentional blink effect was present, the significance of differences between the accuracies at different lags within each condition was tested using a series of paired-sample *t*-tests. Analyses of T2 were restricted to trials with an accurate response on T1. The results indicated that regardless of condition (positive/negative/neutral T1), participants made significantly more errors reporting T2 at lag 2 than at lag 4 and 6, and more at lag 4 than at lag 6. For details please see Table 2.

**TABLE 2:** Significance of differences between accuracies in different conditions of attentional blink task.

**Difference tested**	***M diff* (SD)**	***t* (*df*)**	***p***
T1 positive lag 2 – T1 positive lag 4	−0.32 (1.17)	−19.19 (102)	<0.001
T1positive lag 2 – T1 positive lag 6	−0.35 (0.18)	−19.29 (102)	<0.001
T1positive lag 4 – T1 positive lag 6	−0.03 (0.10)	−2.59 (102)	0.01
T1 negative lag 2 – T1 negative lag 4	−0.30 (0.20)	−15.15 (102)	<0.001
T1negative lag 2 – T1 negative lag 6	−0.35 (0.22)	−16.52 (101)	<0.001
T1negative lag 4 – T1 negative lag 6	−0.05 (0.12)	−3.91 (101)	<0.001
T1neutral lag 2 – T1 neutral lag 4	−0.30 (0.20)	−15.37 (102)	<0.001
T1neutral lag 2 – T1 neutral lag 6	−0.35 (0.22)	−16.44 (102)	<0.001
T1neutral lag 4 – T1 neutral lag 6	−0.05 (0.11)	−4.39 (102)	<0.001

*T1 positive lag 2 – accuracy of responses when T2 is preceded by positive T1 and there are 2 filler words between T1 and T2; T1 positive lag 4 – accuracy of responses when T2 is preceded by positive T1 and there are 4 filler words between T1 and T2; T1 positive lag 6 – accuracy of responses when T2 is preceded by positive T1 and there are 6 filler words between T1 and T2; T1 negative lag 2 – accuracy of responses when T2 is preceded by negative T1 and there are 2 filler words between T1 and T2; T1 negative lag 4 – accuracy of responses when T2 is preceded by negative T1 and there are 4 filler words between T1 and T2; T1 negative lag 6 – accuracy of responses when T2 is preceded by negative T1 and there are 6 filler words between T1 and T2; T1 neutral lag 2 – accuracy of responses when T2 is preceded by neutral T1 and there are 2 filler words between T1 and T2; T1 neutral lag 4 – accuracy of responses when T2 is preceded by neutral T1 and there are 4 filler words between T1 and T2; T1 neutral lag 6 – accuracy of responses when T2 is preceded by neutral T1 and there are 6 filler words between T1 and T2.*

### Life Events, Rumination, Cognitive Biases, and Depression

Logistic regression analysis was conducted to determine which of the analyzed variables were significantly related to depression. Before running the analysis, predictors were converted into z-scores. Because hypotheses were directional, a one-tailed test of significance was used to determine the significance level of the coefficients. Sex was controlled for. The model turned out to be statistically significant, χ^2^(5) = 30.16, *p* < 0.001, Nagelkerke’s *R*^2^ = 0.42. The results indicate that depression is significantly related to life events (*B* = 1.88, *OR* = 6.54, *SE* = 0.65, *p* < 0.01), rumination (*B* = 0.72, *OR* = 2.06, *SE* = 0.32, *p* < 0.05) and memory bias (*B* = 0.52, *OR* = 1.68, *SE* = 0.29, *p* < 0.05), but not to attentional bias (*B* = 0.10, *OR* = 1.11, *SE* = 0.28, *p* = 0.36). The odds of being depressed increase with the distress caused by life events, tendency to ruminate, and with negative memory bias.

### Interplay Between Life Events, Cognitive Factors, and Depression

To test if the tendency to ruminate as well as cognitive (memory and attentional) biases moderate the relationship between life events and depression, logistic regression analyses with interaction terms were implemented. In the first three analyses, the main effects of life events, one of the moderator variables (rumination/attentional bias/memory bias) and their interaction were examined. The last two analyses examined the three-way interaction of life events, rumination and cognitive biases (memory bias/attentional bias) on depression. Prior to conducting the analyses, statistical predictors were converted into z-scores. A one-tailed test of significance was used to determine the significance of main effects as well as the interaction effect in models that tested directional hypotheses. Participants’ sex was controlled for in all interaction analyses.

The results indicated significant effects of interaction between life events and rumination (*B* = 1.48, *SE* = 0.85, *p* < 0.05), as well as between life events and attentional bias (*B* = 1.57, *SE* = 0.88, *p* < 0.05). There was no interaction effect of life events and memory bias on depression (*B* = 0.59, *SE* = 0.52, *p* = 0.13). The three-way interactions (two-tailed) between life events, tendency to ruminate and memory bias (*B* = 0.05, *SE* = 0.75, *p* = 0.94), as well as between life events, tendency to ruminate and attentional bias (*B* = 0.39, *SE* = 0.72, *p* = 0.59) were non-significant. For details see Tables 3, 4.

**TABLE 3:** Analysis of the moderating role of rumination in the prediction of depression from life events.

				**CI 95%**			
				**(two-sided)**		**CI 90%^a^**	
	**Estimate**	***SE***	***OR***	**Lower**	**Upper**	**Lower**	***p***
Intercept	1.10	0.52	3.02	–	–	–	<0.05
Life events	2.03	0.75	7.58	1.74	33.00	2.21	<0.01
Rumination	1.02	0.47	2.76	1.11	6.86	1.29	<0.05
Interaction	1.48	0.85	4.40	0.83	23.38	1.08	<0.05

*^*a*^Since one-tailed tests of significance were conducted separately from 95% two-sided confidence intervals, we reported a 90% lower confidence interval; χ^2^(4) = 26.86, p < 0.001; Negelkerke’s R^2^ = 0.36; OR, odds ratio; one-tailed test of significance was used to determine significance level of predictors; sex was controlled for.*

**TABLE 4:** Analysis of the moderating role of negative attentional bias in the prediction of depression from life events.

				**CI 95%**			
				**(two-sided)**		**CI 90%^a^**	
	**Estimate**	***SE***	***OR***	**Lower**	**Upper**	**Lower**	***p***
Intercept	0.85	0.	2.34	–	–	–	0.07
Life events	1.93	0.66	6.87	1.90	24.86	2.33	<0.01
Attentional bias	0.90	0.52	2.45	0.88	6.82	1.04	<0.05
Interaction	1.57	0.88	4.80	0.86	26.71	1.14	<0.05

*^*a*^Since one-tailed tests of significance were conducted separately from 95% two-sided confidence intervals, we reported a 90% lower confidence interval; χ^2^(4) = 24.47, p < 0.001; Negelkerke’s R^2^ = 0.34; OR, odds ratio; one-tailed test of significance was used to determine significance level of predictors; sex was controlled for.*

**FIGURE 1:**
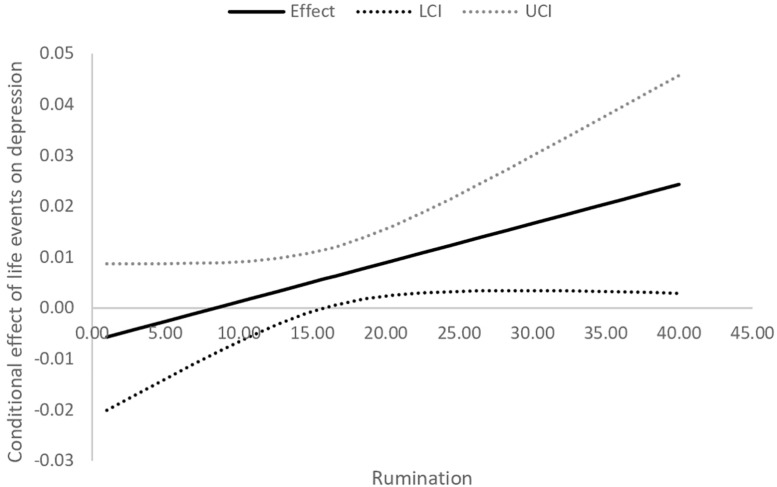
The Johnson-Neyman graph for the model relating depression to life events, rumination and their interaction. The effect of life events on depression is significant only for close-to-average and elevated levels of rumination.

**FIGURE 2:**
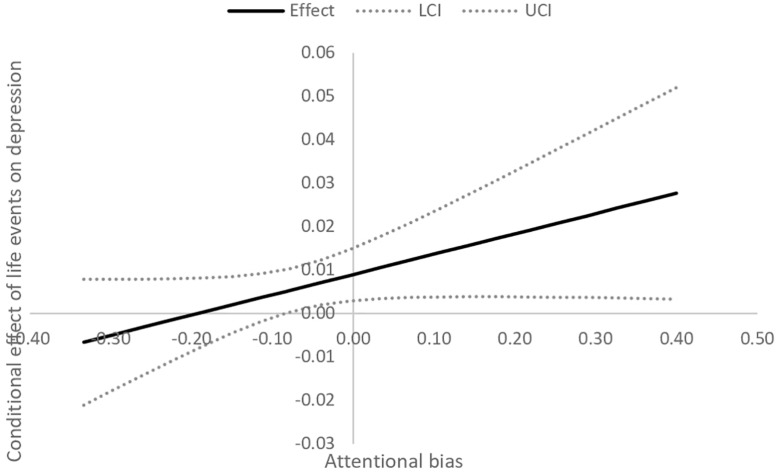
The Johnson-Neyman graph for the model relating depression to life events, attentional bias and their interaction. The effect of life events on depression is significant only for close-to-average and elevated levels of negative attentional bias.

The Johnson–Neyman technique (Johnson and Fay, 1950) was utilized to probe significant interaction effects. This approach searches for values of moderator variables that make a significant effect of the predictor variable on the dependent variable. It is described as a global “floodlight” technique, in contrast to local “spotlight” methods such as traditional simple slope analysis (Spiller et al., 2013). Figures 1, 2 show the Johnson–Neyman graphs for the significant moderation models. The effect of life events on depression is significantly different than 0 for values of rumination higher than 15 points (*z* = -0.54) and values of attentional bias indicator exceeding -0.10 (*z* = -0.54). In other words, life events are associated with a higher probability of being classified as depressed, but only in the case of individuals with a close to average and elevated level of negative attentional bias/tendency to ruminate. This link is not significant for individuals with low levels of these cognitive vulnerability factors.

## Discussion

When the general model with all the predictors was tested, the results indicated that stressful life events together with individual tendency to ruminate and negative memory bias were significantly related to depressive symptoms in young adults. This result is generally in line with many earlier studies (e.g., Matt et al., 1992; De Raedt and Koster, 2010; Disner et al., 2011; Lester, 2014; Bastin et al., 2015; Connolly and Alloy, 2017), and also suggests that these three factors may work together in generating symptoms of depression. However, we did not confirm Koster’s results: attentional bias was not significantly linked to depression in the current study. A plausible explanation of the differences in the obtained results is dissimilarities in the selection criteria of participants. An unconstrained group of students participated in our study, whereas in Koster’s investigation this was a carefully selected group of dysphorics. A more direct comparison of both groups is hampered by the use of different tools to measure depressive symptoms (BDI vs. CES-d). Also, Koster et al. (2009) did not take into account the negative life events we examined, so it is difficult to say to what extent the differences between dysphorics and non-dysphorics obtained in their study could be related to this variable.

It was also hypothesized that the relationship between stressful life events and depressive symptoms is modified by individual tendency to ruminate, negative memory bias, and attentional bias. These effects were confirmed in the case of rumination and attentional bias, but not memory bias. Three-way interactions between stressful experiences, habitual rumination and cognitive biases were also non-significant. While the interactive effect of stress and rumination has been shown in many earlier studies (e.g., Bastin et al., 2015; Hamlat et al., 2015; Connolly and Alloy, 2017; Shapero et al., 2017), it seems that negative attentional bias may play a similar role in the relationship between stressful life events and depressive symptoms: the stronger the effect of emotional attentional blink, the stronger the relationship between negative life events and symptoms of depression. The obtained results are in line with cognitive models of depression that emphasize the role of selective attention for negative information in the formation of depressive symptoms (e.g., Beck, 2008, 2011); and suggest that cognitive biases, i.e., negative attentional bias, constitute vulnerability factors which make individuals more likely to become depressed following stressful negative events. Although the status of attentional emotional blink is not fully established in the literature and one could possibly treat it as simply another symptom of depression, the results of the present study negate such a conclusion since it works only as a moderator, not as a simple statistical predictor.

Memory bias turned out to be related to depressive symptoms; this confirmed our hypotheses and the results of earlier studies (Matt et al., 1992), which showed that depressive symptoms are associated with improved recall of negative information or with the lack of positive recall asymmetry that is often found in the non-depressed. However, we did not obtain evidence supporting our hypothesis that memory bias modifies the stress–depression relationship. This finding seems to be inconsistent with the cognitive theory of depression, which suggests that negative experiences influence depression through the activation of negative schemas (e.g., Beck, 1976). However, the nature of the stress that activates cognitive schemas is not well defined and conceptualizations of stress differ within vulnerability–stress models and research. It is possible, therefore, that the life stressors that occurred during the 6 months preceding the study were not sufficient for the additional activation of memory bias.

The specific nature of the stress inventory used in our study may also partially explain the lack of evidence for the predicted moderating role of memory bias in the stress–depression relationship. In the stressful life events scale, the participants were asked to report the events which had happened to them in the last 6 months and evaluate how stressful they were. It cannot be ruled out that people with a higher level of depressive symptoms were more likely than people in a better mood to recall and report negative and difficult life events that might have affected the studied relationship between memory and stress. However, it also possible that memory and attentional bias influence depressive symptoms differently. Negative memory bias may contribute to depression regardless of recent stressful experiences, while attentional bias influences the development of depressive symptoms only in individuals who are exposed to stressors. The material recalled from memory may be related to negative life events that took place earlier than the period covered by the study. Moreover, even life events that are not very “objectively” negative may contribute to the appearance of depressive symptoms if recalled often and ruminated on. This means that biased memory processes, but not attentional ones, which rely more on external cues, may contribute to depressive symptoms relatively independently of recent negative life stressors.

It is also possible that symptoms of depression may be affected by cognitive biases not separately but in a collective manner, as postulated by some authors (Everaert et al., 2012; Everaert et al., 2015). According to the combined cognitive bias hypothesis, cognitive biases operate in concert and their combination has a greater impact on disorders than each of them taken in isolation (Hirsch et al., 2006). However, additional analyses conducted on the data collected in the present study showed that the two-way interactive effect of memory and attentional bias on depression was non-significant (*B* = 0.14, *SE* = 0.27, *p* = 0.60), thus suggesting that they influence depression not conjointly but independently from each other. Also, there was no significant correlation between attention and memory bias measures. This may seem quite surprising given the fact that the cognitive model predicts mood-congruent biases at several stages of information processing. On the other hand, a similar lack of a connection between attention and memory bias has already been observed in other studies (Gotlib et al., 2004a; Vrijsen et al., 2014). This may indicate that these biases reflect distinct processes and it is important to distinguish them in order to better understand the mechanism of depression.

Although, in principle, all cognitive models of depression are vulnerability–stress models, research based on these models explores the role of stress to varying degrees. Stress is sometimes taken into account in studies on negative cognitive style, hopelessness, dysfunctional attitudes, and rumination (e.g., Dixon et al., 1993; Dykman and Johll, 1998; Stange et al., 2013; Shapero et al., 2017). However, in almost all of these studies, cognition is assessed using self-report measures. Many such studies, both cross-sectional and prospective, suggest that these cognitive styles predict depression following stressful events, but the underlying cognitive mechanisms are less precisely established (e.g., Beevers and Carver, 2003). Experimental performance-based measures like memory tasks or the RSVP task may allow the underlying cognitive biases at different stages of information processing to be explored. In contrast to self-report measures, these methods do not rely on introspection; instead, they examine processes that elude self-description. Although stress is sometimes taken into account as a factor activating cognitive schemas in areas of research in which performance-based measures of cognitive biases are used, this is achieved mainly by mood induction procedures (Timbremont and Braet, 2004; Scher et al., 2005). The roles of stress conceptualized as recent stressful life events is usually overlooked.

### Limitations

According to the obtained results, stressful life events, the tendency to ruminate, and cognitive biases may be considered only as factors contributing to the development of depression symptoms, but not to clinical depression. The study lacked a standardized interview conducted by a clinician to confirm the presence of psychiatric conditions and use of medication. Participants were a non-clinical sample who reported not having clinically diagnosed depression, and those who were assigned to the depression group simply had higher scores on the CES-D scale. The possibility of applying the obtained results to clinical depression is therefore limited to some extent, however, considering the dimensional approach to psychopathology, it seems to be justified.

The cross-sectional design of the study limits conclusions regarding causality. Although the “diathesis–stress” model and cognitive theories suggest that cognitive distortions and negative life events are risk factors for developing depression, it cannot be ruled out that they are in fact consequences of the presence of depressive symptoms, as postulated by the stress generation model (e.g., Hammen, 1991). Since according to Eberhart et al. (2011) the two mechanisms are not mutually exclusive, a tentative conclusion can be drawn that depressive participants could to some extent contribute to the occurrence of the reported stressful events that were person dependent. The distinction between dependent and independent events was not included in the analysis because such a distinction may be difficult in the case of some events (as, for example, in the case of serious illness or being a victim of a crime). Also, the design of the current study makes it impossible to determine whether cognitive vulnerability factors moderate the link between stress and depression, or whether it is the other way around, as has been assumed previously in some research (e.g., Connolly and Alloy, 2017). Therefore, the results obtained in the study should be treated as preliminary; to address this issue, future studies should implement a longitudinal design.

We used a cumulative index of stress that included a number of events and individual evaluation of events. While it could be possible that cognitive biases influenced this measure, results of additional analyses using only the number of events showed analogous relationships between variables. Moreover, we measured stressful life events with a questionnaire created by us for the purpose of the study. Even though it proved to have good internal consistency and – in line with the results of previous empirical research concerning rumination – its scores were associated significantly and positively with depression symptoms, we did not check its convergent validity with other existing measures of stressful life events. It therefore restricts to a certain degree the possibility of comparing the current findings with those derived from previous research using other forms of assessments. Similarly, the convergent validity of the Rumination Questionnaire by Baryła and Wojciszke (2005) with other measures of rumination has not been assessed.

Participants were tested in group sessions. Although the utmost care was taken to ensure that the conditions in each session were as comparable as possible and the testing took place in a sound-attenuated laboratory, the influence cannot be ruled out of confounders such as distractors in the performance of cognitive tasks or social desirability effects in self-reported measures. Also, data was obtained from a very selective and homogeneous sample of university and high school students, which clearly limits generalization of the results. However, it should be mentioned that we chose the group of young adults for a few reasons. Firstly the cognitive schemas that modify the interpretations of experiences are fully established and relatively stable. Secondly, since it is a transitional period in life (from young to adult) individuals are more frequently exposed to stressful life events. Thirdly, the biological determinants of depression that occur during adolescence are significantly less pronounced during early adulthood, thus the psychological determinants of depression can be studied more reliably.

There were far more female participants than male; thus, there is the risk that the obtained results concern women more than men. Although the conducted analyses did not show sex to be significantly related to depressive symptoms when analyzed together with cognitive biases, rumination and stressful life events further studies should use groups with more balanced numbers of participants of both sexes.

In our research we decided to divide the sample basing on the CES-D score into two groups consisting of depressed and non-depressed individuals. This approach has both advantages and disadvantages. On the one hand it is common practice in clinical studies to dichotomize continuous outcome variables, especially when the range of the variable has distinct clinical significance because therapeutic decisions, procedures and interventions are usually based on thresholds and cut-off points rather than on continuous values. On the other hand, as mentioned earlier, the sample in the current study was rather homogenous and characterized by non-clinical distributions of depression level, which may be an argument for using absolute CES-D scores instead of a dichotomized variable in the analyses. It should be mentioned that when applying linear instead of logistic regression analysis to test our hypothesis, the interaction effects did not turn out to be significant. The possible reason for this inconsistency between analyses may be that in the presence of unknown additive contamination errors, dichotomization of the response variable sometimes produces a better result in statistical analysis than using absolute scores (Shentu and Xie, 2010).

Finally, one of the possible limitations of our research in the context of establishing the relationship between stress and memory processes is that the life events scale relies on memory processes. Another method of studying stressful life events that would not be affected by possibly biased memory processes of the subjects might be more suitable; for example, an ecological momentary assessment design (Connolly and Alloy, 2017).

## Conclusion

The study provided additional data concerning the relationship between life events, cognitive factors and depression. Our findings indicate that the link between stressful life events and depression is visible only among individuals who exhibit specific cognitive vulnerability. In particular, people showing attentional bias toward negative stimuli, as well as individuals with a tendency to ruminate, may be prone to negative effects of stressful experiences. Moreover, obtained results tentatively suggest that memory and attentional bias may play a different role in depressive symptoms formation, the first one changing the effect that negative life events have on people, the second one influencing depression independently from negative experiences. It is also worth mentioning that the moderating effect of attentional bias on the stress–depression symptoms relationship is – to the best of our knowledge – the first such result obtained using the RSVP task.

It is worth considering the possible clinical implications of our study. The results, which suggest that cognitive factors moderate the effect of negative life events on the onset of depressive symptoms, are in agreement with the cognitive-behavioral model of depression. Thus, it may be worthwhile to design preventive cognitive training programs aimed specifically at modifying cognitive biases and rumination tendencies in situations of the accumulation of negative life events (e.g., Browning et al., 2010). Such interventions could work as a form of cognitive vaccine, preventing an increase of depressive symptoms in the face of stressors, and thus reducing the likelihood of clinical depression.

## Data Availability Statement

The datasets generated for this study are available on request to the corresponding author.

## Ethics Statement

The studies involving human participants were reviewed and approved by Komisja Etyczna Instytut Psychologii Uniwersytet Jagiellonski. The patients/participants provided their written informed consent to participate in this study.

## Author Contributions

WŁ, AB, and JK designed the study and interpreted the results. WŁ wrote the first draft of the manuscript. JK and JŁ-P collected the data for the study. JK ran the statistical analyses. JK, AB, and JŁ-P edited the first draft of the manuscript. All authors approved the final version of the manuscript.

## Conflict of Interest

The authors declare that the research was conducted in the absence of any commercial or financial relationships that could be construed as a potential conflict of interest.
